# A prospective observational study of community acquired pneumonia in Kenya: the role of viral pathogens

**DOI:** 10.1186/s12879-021-06388-x

**Published:** 2021-07-23

**Authors:** Jamila Nambafu, Mary Achakolong, Fridah Mwendwa, Jumaa Bwika, Felix Riunga, Samuel Gitau, Hanika Patel, Rodney D. Adam

**Affiliations:** 1 Department of Medicine, Aga Khan University, Third Parklands Rd, Nairobi, Kenya; 2 Department of Pathology, Aga Khan University, Third Parklands Rd, Nairobi, Kenya; 3 Department of Radiology, Aga Khan University, Nairobi, Kenya

**Keywords:** Pneumonia, Viral pneumonia, Community acquired pneumonia, Sub-Saharan Africa, Kenya, Influenza, Tuberculosis, *Pneumocystis jirovecii*, Diabetes, HIV

## Abstract

**Background:**

Lower respiratory tract infections continue to contribute significantly to morbidity and mortality across all age groups globally. In sub-Saharan Africa, many studies of community acquired pneumonia in adults have focused on HIV-infected patients and little attention has been given to risk factors and etiologic agents in an urban area with a more moderate HIV prevalence.

**Methods:**

We prospectively enrolled 77 patients admitted to a 280 bed teaching hospital in Kenya with radiographically confirmed community acquired pneumonia from May 2019 to March 2020. The patients were followed for etiology and clinical outcomes. Viral PCR testing was performed using the FTD respiratory pathogen-21 multiplex kit on nasopharyngeal or lower respiratory samples. Additional microbiologic workup was performed as determined by the treating physicians.

**Results:**

A potential etiologic agent(s) was identified in 57% including 43% viral, 5% combined viral and bacterial, 5% bacterial and 4% *Pneumocystis*. The most common etiologic agent was Influenza A which was associated with severe clinical disease. The most common underlying conditions were cardiovascular disease, diabetes and lung disease, while HIV infection was identified in only 13% of patients. Critical care admission was required for 24, and 31% had acute kidney injury, sometimes in combination with acute respiratory distress or sepsis.

**Conclusion:**

Viruses, especially influenza, were commonly found in patients with CAP. In contrast to other studies from sub-Saharan Africa, the underlying conditions were similar to those reported in high resource areas and point to the growing concern of the double burden of infectious and noncommunicable diseases.

**Supplementary Information:**

The online version contains supplementary material available at 10.1186/s12879-021-06388-x.

## Background

According to the Global Burden of Diseases (GBD), lower respiratory tract infections (LRIs) were reported to have caused 2.7 to 2.8 million deaths per year from 2005 to 2015 and close to half of these were among adults [1]. Sub-Saharan Africa (SSA) is disproportionately affected by a higher burden of lower respiratory tract infections which affect a much younger population when compared to the North American and European cohorts [2].

Studies on viral etiology of community acquired pneumonia (CAP) among adults within the sub-Saharan African region are scarce and where available, are often combined with pediatric cohorts [3]. In addition, some of the reports are from settings with very high HIV and tuberculosis prevalence. The high prevalence of HIV-associated comorbidities, lack of control cohorts and absence of standard definitions make it difficult to consistently determine and define the overall burden of viral lower respiratory disease or characterize disease by etiology [3]. In contrast, Kenya had an estimated HIV prevalence of 4.9% in 2017 (6.1% in Nairobi), which places it above most countries in other continents but substantially lower than many countries of sub-Saharan Africa [4]. The prevalence of non-communicable diseases (NCDs) such as obesity and diabetes mellitus has been increasing in sub-Saharan Africa, creating new potential risk factors for CAP. These conditions have been associated with increased susceptibility to viral infections especially with influenza among diabetic patients [5–7]. Having recognized this inimical synergy, some countries, such as South Africa, have designed and rolled out specific health care guidelines for the management of CAP among susceptible groups including those with diabetes mellitus [8].

Before the onset of the COVID-19 pandemic, influenza was the most common cause of severe life threatening and vaccine preventable viral lower respiratory disease among adults worldwide [9–12]. In addition, numerous other viral pathogens are recognized as important causes of lower respiratory tract infections (LRTI). Respiratory syncytial virus (RSV) is second to Influenza as a cause of LRTI and shows winter seasonality similar to influenza in temperate climates [13–16]. Human metapneumovirus and human parainfluenza virus are within the same family of viruses as RSV and cause influenza-like symptoms in high-risk populations such as the elderly and the immunocompromised [17–20]. In addition, adenoviruses [21] and rhinoviruses [11] are sometimes implicated as causes of LRTI.

We have therefore investigated the roles of these viral pathogens as causes of CAP in a setting with moderate levels of HIV and a growing prevalence of diabetes, hypertension and other NCDs.

## Methods

### Setting

The Aga Khan University hospital is a teaching hospital in Nairobi with 280 beds that include 33 adult critical care beds and over 20 medical specialties. The hospital has been accredited by the Joint Commission International since 2013 and the laboratory has been accredited by SANAS since 2011 [22] and by the College of American Pathologists since 2018.

### Study design and definitions

We conducted a prospective observational study of inpatients with community acquired pneumonia from May 1st, 2019 until March 13th, 2020. The enrollment was planned for a complete year but was terminated in March due to the COVID-19 pandemic. The first case in Kenya was reported on March 13, 2020. This series therefore represents the spectrum of pre-COVID-19 pneumonia.

Patients over 18 years of age admitted with suspected community acquired pneumonia were approached for enrollment if they had a new infiltrate on pulmonary imaging with compatible symptoms such as cough, fever, breathlessness, and production of sputum. Patients were excluded if they had a pulmonary infection within 6 weeks of presentation or chronic lung disorders judged to be significant enough to compromise interpretation of the diagnosis or outcome of pneumonia. Chest images (chest radiograph or CT Scan) obtained within 72 h of admission were used to identify changes consistent with lower respiratory infection findings, including opacities, effusions, consolidations, and other findings. For patients where both radiograph and CT were done, analysis was based on CT findings. All enrolled patients were followed up for critical care admission within 30 days and for discharge status (alive/dead) or death within 30 days.

Acute kidney injury (AKI) was defined using the KDIGO criteria [23]. Sepsis was defined using the Sepsis 3 criteria [24] as life-threatening organ dysfunction caused by a dysregulated host response to infection using the Sequential Organ Failure Assessment (SOFA) to identify organ dysfunction (Ranieri et al., 2012). PSI [25] and CURB65 [26] are validated scores for mortality prediction (Shah BA et al., 2010) [27, 28] due to CAP and were used as described. The PSI score is calculated from the following parameters; age, sex, nursing home resident, neoplastic disease, liver disease history, CHF history, cerebrovascular disease history, renal disease history, altered mental status, respiratory rate ≥ 30 breaths/min, systolic blood pressure < 90mmhg, temperature < 35 C or > 39.9 C, pulse ≥125 beats/min, pH < 7.35, Blood urea ≥30 mg/dL or ≥ 11 mmol/L, Sodium < 130 mmol/L, glucose ≥14 mmol/L, hematocrit < 30%, partial pressure of oxygen < 60 mm Hg and pleural effusion on Xray. PSI scores of 1 to 3 are classified as low risk, while PSI 4 to 5 are either moderate or high risk. CURB65 is derived from the following: Confusion, blood urea > 7 mmol/l, respiratory rate ≥ 30, systolic blood pressure < 90 mm Hg diastolic blood pressure  ≤  60 mm Hg and Age ≥ 65 years. CURB65 scores of 0 to 2 are considered low risk; 3 is deemed to be moderate risk, and 4 to 5 are regarded as high risk.

The WHO definition for severe acute respiratory infection (SARI) was used which includes a history of fever or measured fever of ≥38 C° with onset in the last 10 days, cough and a requirement for hospitalization [29].

### Microbiological workup

After informed consent was obtained, nasopharyngeal swabs were collected by a trained clinician using Sterile Snappable Polystyrene & Viscose Amies Swabs (Deltalab, Barcelona, Spain). Bronchoalveolar lavage (BAL) specimens were used when available. Samples were stored at − 80 °C until testing. Nucleic acid extraction was done using QIAamp Viral RNA Minikit (Qiagen, Germany) as per manufacturer’s instructions and reverse transcriptase-PCR was done in a Rotor-gene Q thermal cycler (Qiagen, Germany) using the FTD Respiratory pathogens 21 test (Resp21; Fast Track Diagnostics, Luxembourg). Resp21 detects the following pathogens: influenza A virus (unknown subtype); influenza A(H1N1) virus (swine-lineage); influenza B virus; human rhinovirus; human coronaviruses NL63, 229E, OC43 and HKU1; human parainfluenza viruses 1, 2, 3 and 4; human metapneumoviruses A/B; human bocavirus; human respiratory syncytial viruses A/B; human adenovirus; enterovirus; human parechovirus and *Mycoplasma pneumoniae.* The first 73 patients were tested using the Resp21 test. The same reagents were not available for the last four patients, so their samples were tested for Influenza A, H1N1, Influenza B, RSV A and RSV B using Realstar Influenza Screen & Type RT-PCR Kit 4.0 and Realstar RSV RT-PCR Kit 3.0 respectively (both produced by Altona Diagnostics, Hamburg, Germany).

Other microbiological evaluations were performed as requested by the treating physician and were not influenced by the study personnel. Microbiological reports that were considered include sputum Gram stains and cultures and blood cultures as well as influenza or RSV rapid antigens. *Pneumocystis* PCR is highly sensitive but not highly specific, so patients with positive *Pneumocystis* PCR were evaluated according to clinical characteristics and cytologic findings.

## Results

### Demographic profile

**Fig. 1:**
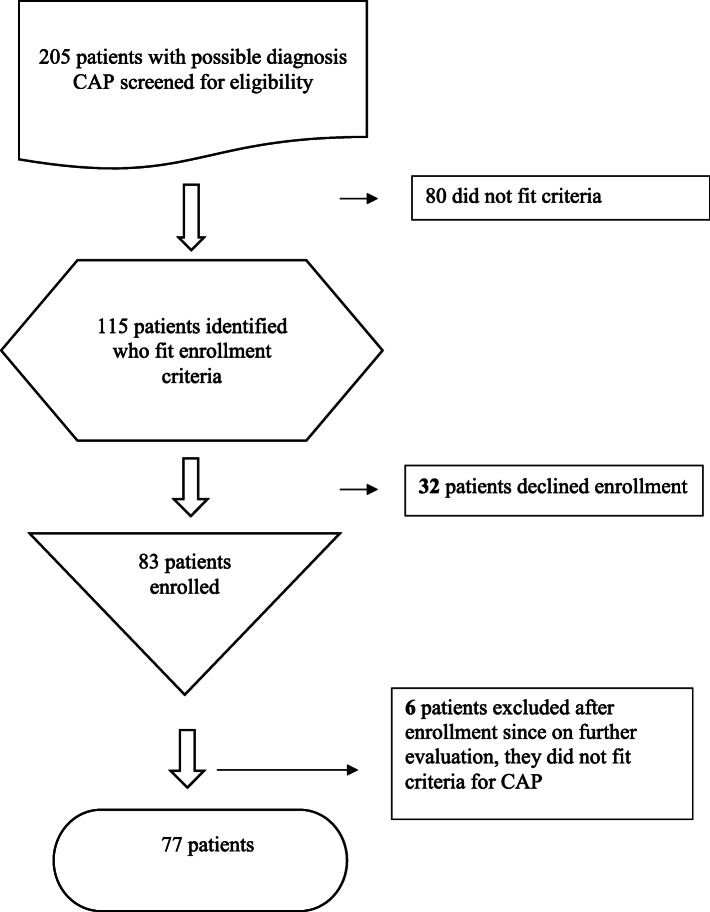
Flow chart of patient recruitment

A total of 77 patients fitting the inclusion criteria were enrolled in the study (Fig. 1). The median age was 54.5 (IQR 38–71.5) years and 57% were male. Eighty-three percent had at least one pre-existing condition, including cardiovascular disease (43%), followed by lung disease (22%), diabetes mellitus type II (22%), and HIV (13%) (Table 1). Almost half the study population (46%) had used an antibiotic before admission, with the most frequently used being aminopenicillins (amoxicillin-clavulanate), macrolides (azithromycin) and cephalosporins (cefuroxime or ceftriaxone).

**Table 1:** Patient characteristics

Patient Category No. (%) of patients *N* = 77/ (%)			
Age (Mean, SD)	54.0 (±16)	**Signs**	
60 years old or more	33 (42.9)	RR ≥ 30/min	6 (7.8)
**Gender**		Heart rate ≥ 125/min	13 (16.9)
Male	44 (57.1)	Temperature ≥ 39.5 or ≤ 35	13 (16.9)
**Comorbidities**		SpO2 ≤ 92	38 (50.6)
CVD	32 (41.6)	SBP < 90 mmHg	8 (10.4)
Diabetes	23 (29.9)	**Laboratory findings**	
Lung disorders	26 (33.8)	WBC ≥ 10,000	34 (44.2)
CKD	9 (11.7)	Hemoglobin ≤10 g/dl	6 (7.8)
HIV infection	10 (13.0)	Platelets< 150,000	17 (22.1)
Prior Antibiotics	35 (45.5)	BUN (Urea) > 7 mmol/l	16 (20.8)
**Lifestyle**		Serum sodium ≤130	10 (13.0)
Active Smoker	5 (6.5)	**Imaging findings**	
Alcohol	17 (22.1)	Ground glass opacities	27 (35.1)
		Other Opacities	14 (18.2)
**Symptoms**		Multiple findings^a^	10 (13.0)
SARI	61 (79.2)	Consolidations	23 (29.9)
Fever	75 (97.4)	Effusions	2 (2.6)
SOB (dyspnea)	60 (77.9)	Cavity	1 (1.3)
Chest pain	62 (80.5)	Bilateral findings^b^	27 (35.1)
Malaise & fatigue	39 (50.6)	**Case admissions**	
Sputum	18 (23.4)	Non-Critical	53 (68.9)
Weight loss	21 (27.3)	Critical Care	20 (26.0)
Wheezing	18 (23.4)	Critical Care and Intubated	4 (5.2)

*CKD* chronic kidney disease

*CVD* cardiovascular disease (hypertension, arrhythmias and heart failure)

*SOB* Shortness of breath

*SARI* Severe Acute Respiratory Illness; defined as a history of fever or documented temperature of 38 °C and above and cough requiring hospitalization within the last 10 days

^a^Multiple findings included reticulonodular changes and peribronchial cuffing that could not be placed into any of the above categories

^b^Majority of the bilateral findings were opacities (13/27, 48%)

Most of the enrolled patients had complaints of cough, fever, difficult breathing, chest pain, and malaise, all of which were present in at least half the patients. Seventy-nine percent fit criteria for severe acute respiratory illness (SARI).

### Etiologies

Seventy-five of the 77 enrolled patients had a nasopharyngeal swab obtained for viral PCR using the Resp21 assay as part of the study protocol for 71 patients, while four were tested by PCR using the Altona influenza and RSV tests. Other tests were done as determined by the admitting physician and included blood cultures (60) and sputum [30]. Bronchoscopy was done in seven. A potential etiology was identified in 44 (57%) of the patients (Table 2).

**Table 2:** Etiologic agents of pneumonia

Type of infection and agents identified	***n*** = 77 (%)
None	33 (42.9)
Viral pathogen alone (single agent)	22 (28.6)
Viral pathogen alone (multiple agents)	11 (14.3)
Bacterial pathogen alone	4 (5.2)
Viral-bacterial co-infection	4 (5.2)
*P. jirovecii*	3 (3.9)
**Viral agents:**	
Influenza A	18 (23.4)
Rhinovirus	18 (23.4)
Adenovirus	6 (7.8)
Enterovirus	4 (5.3)
Parainfluenza	3 (3.9)
Respiratory Syncytial Virus	2 (2.6)
Parechovirus	2 (2.6)
Human Metapneumovirus	1 (1.3)
**Bacterial agents:**	
**Blood culture (*n*** **= 60)**	
*E. coli*	1 (1.3)
S. *Typhi*	1 (1.3)
*S. pneumoniae*	2 (2.6)
**Bronchial wash culture (*n*** **= 7)**	
M. *tuberculosis*	1 (1.3)
*K. pneumoniae*	1 (1.3)
**Sputum (*n*** **= 39)**	
M. *tuberculosis*	1 (1.3)
S. *pneumoniae*	1 (1.3)

Viral agents were identified in 37 patients (48%) and for 33 of these patients, the only pathogen identified was a virus, while the other four had *Mycobacteria tuberculosis* or a pyogenic bacterium identified. Most of the viral pathogens were identified from nasopharyngeal swabs, but three of four oropharyngeal swabs and two of the three BAL samples subjected to PCR had viruses identified. For the other four patients with BAL performed, PCR was not performed on the BAL fluid, but was done on nasopharyngeal swabs obtained upon enrollment and one had a virus identified.

The most commonly identified virus that is consistently recognized as a lower respiratory pathogen was Influenza A, which was found in 18 patients. It was the sole pathogen in 11 patients. It was found with one or more other viruses in three patients, and with bacterial infection in one patient who had pneumococcal bacteremia. Diabetes mellitus was the most common comorbid condition among patients with Influenza A (9 of 18; 50%) and was found more commonly in those with influenza than in those without (14 of 57; OR 3.07, 95% CI 1.02–9.25, *p* = 0.046). Seven of the patients with influenza identified by PCR also had Influenza rapid antigen testing done with only two of these positive, indicating poor performance of the rapid antigen test.

**Fig. 2:**
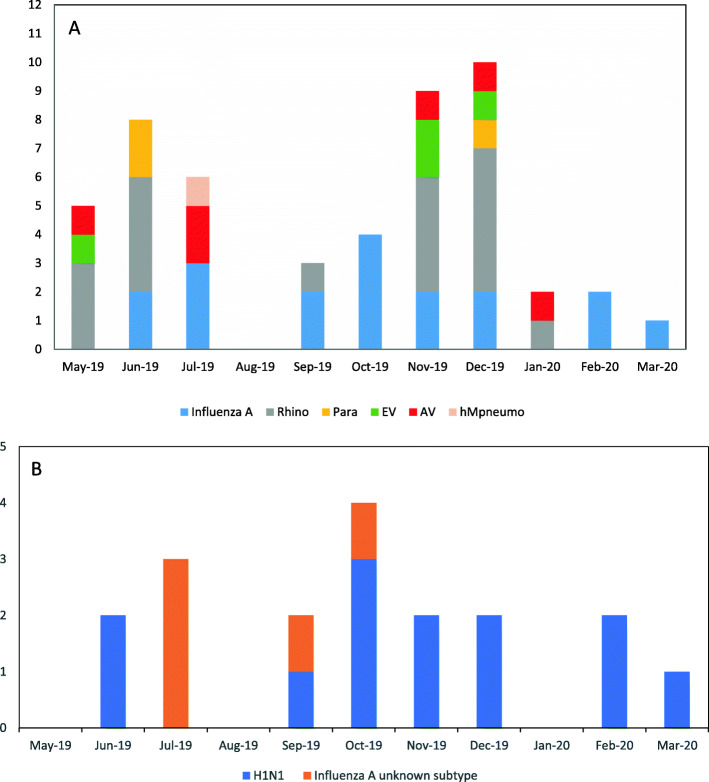
Viruses identified; the number on the left indicates the number of cases. **A** Virus isolation by month; May 2019 to March 2020. **B** Influenza by month

Rhinoviruses were found in 18 patients, but in all but four, were found with other viruses. For the four patients with rhinovirus as the only potential pathogen, we included it as a cause of pneumonia while recognizing its role in LRI as being controversial. Other viruses that were found included adenovirus, enteroviruses, parainfluenza, RSV, parechovirus and human metapneumovirus. These viruses were frequently found in combination. It is notable that the two patients with adenovirus as the sole agent presented with temperatures above 40 °C. Viruses were isolated from patients throughout the 10 months of the recruitment period for total viruses (Fig. 2a) and for influenza, specifically (Fig. 2b).

Pyogenic bacteria were identified as pulmonary pathogens in seven patients; four with positive blood cultures and three with potential pathogens from sputum specimens (Table 2). In addition, two of the patients had positive AFB smears followed by positive cultures for *M. tuberculosis* complex.

Of the 10 patients with HIV, three patients had *Pneumocystis* pneumonia (PCP). These patients had CD4 counts of 4, 22 and 340 cells/mm^3^. One of the patients with PCP had coinfection with both rhinovirus and parainfluenza virus 4. The seven HIV positive patients without PCP included two with influenza, one with rhinovirus-Klebsiella co-infection and another with bacteremic pneumococcal pneumonia. No pathogen was identified among three patients within this cohort and neither of the two patients with TB presenting as CAP was HIV-infected.

### Outcomes

In this study, a low risk PSI score of 1–3 was recorded in 73% of the patients. In the current study, 40% of the patients had a CURB65 score of 0 and none had the high risk scores of 4 to 5 despite the number of patients over 65 years (which adds one point to the score). Despite the low PSI and CURB65 scores, a significant number of patients (34; 43.6%) patients had at least one complication (Table 3). The most common was acute kidney injury (31%), which was found alone in 22% and in combination with ARDS and/or sepsis in another 9%. In addition, 30% percent of the admissions were to critical care units. The average length of hospitalization was 7.4 days, though those in intensive care units spent a mean of 8 days. Two (3%) of the study patients died within 30 days, seven (9%) had readmission within the same period.

**Table 3:** Pneumonia severity indices and level of care, complications and outcome

Level of care	Total	PSI					CURB65				
		1	2	3	4	5	0	1	2	3	4/5
Ward	53	11	24	6	11	1	25	19	7	2	0
Critical care	20	1	7	4	3	4	6	6	5	2	0
Critical + vent	4	0	1	2	1	0	0	2	1	1	0
**Complications**											
AKI	22	2	7	3	6	4	4	6	8	4	0
AKI with RF	2	0	0	1	0	1	0	1	1	0	0
RF	4	0	3	0	1	0	2	1	1	0	0
**Outcome at 30 days**											
Mortality	2	0	1	1	0	0	1	1	0	0	0
Readmission	7	0	1	1	3	2	1	1	4	1	0

The morbidity associated with influenza was greater than that for other viruses or nonviral pneumonia as determined by the number of patients requiring critical care treatment. Patients with influenza were more likely to require critical care than those with other viral pneumonias (61%; OR 5.32, 95% CI 1.72–16.49, *p* = 0.004). In contrast, 21% of those with other viruses not including influenza and 22% of those with no virus identified required critical care.

### Characteristics of the patients that died within 30 days

Two patients died within 30 days of enrollment, both of whom had viral pneumonias. One was a 46 year-old female with diabetes mellitus and hypertension with Influenza A and adenovirus complicated by severe ARDS who died from respiratory failure. The other patient was a 59 year-old female with diabetes mellitus and multiple myeloma. She had parainfluenza pneumonia and was managed on the non-critical ward and discharged after 7 days of hospitalization. She died 1 day after discharge.

## Discussion

This study is one of the few prospective studies of CAP in adults in SSA. Our study focused on routine viral testing accompanied by testing for other pathogens as ordered by the treating physicians. With this combination of testing, a least one potential pathogen was identified in 57% of the study population including viruses in 48%, pyogenic bacteria in 9%, PCP in 5% and TB in 3%. The percentages add up to more than 57% because of the patients with coinfections. Influenza A was the most common pathogen identified in this study, found in 18 patients (24% of total); in 10 as the only pathogen identified and eight in combination with bacteria or other viruses. The original aim of the study was to recruit patients for a full year to allow a more accurate assessment of seasonality; however, when the first COVID-19 case was identified in Kenya in mid-March, recruitment was halted at 10 months, limiting our conclusions about seasonality. However, cases occurred throughout the 10 months of observation with no clear seasonal trend and this finding compares with CDC Surveillance data from Kenya between 2007 to 2013 [31]. Other studies have reported variable trends including May–July peaks in South Africa [12] and January–February peaks in Kenya [9]. The predominance of H1N1 (72%) in our study contrasts with the predominance of H3N2 in the northern hemisphere (Radin et al., 2012), but is consistent with data from east Africa demonstrating seasonal and year to year variability in the prevalence of H1N1 [32, 33].

Previous retrospective and prospective studies on pneumonia in SSA have focused predominantly on bacterial pathogens. A study from Kenya in 2000 reported *S. pneumoniae* as the most common pathogen, accounting for nearly 50% of cases [34]. In the aforementioned study, antibody testing was used for viral pathogens allowing the identification of influenza as the etiologic agent in 5% of patients. Their study was performed in public hospital with a high percentage of HIV-infected patients reported as 52% of 281 participants and half of the *S. pneumoniae* cases were in HIV-infected patients. We note that in the current series, three of the 10 HIV-infected patients had PCP as causes for their CAP, while one had pneumococcal bacteremia and none had TB. Other recent studies have demonstrated a wide range of pathogens. A study in Malawi with an HIV positive adult cohort of 94% (48/51) found that 29% bacterial infection, 27% PCP, 22% pulmonary TB and only 6% had viral infections [35]. Another study from Cameroon comparing only clinical parameters among HIV-negative and positive adults with pneumonia reported a rate of HIV infection of 59% (62/106) (Yone et al. 2012). Several other studies among patients with pneumonia have alluded to the high prevalence of HIV and very little has been mentioned about NCDs [34–38].

As in many studies throughout the world, males were more common than females [37], Underlying conditions known to confer increased risk for CAP were identified in 83% of patients of which the most common were cardiovascular disease, diabetes and chronic pulmonary disease. A study done in Nigeria [39], revealed 38.8% of patients with CAP had at least one NCD. Studies from high and middle income countries have also shown that NCDs are common in patients presenting with pneumonia [30, 40, 41] and are linked to increased morbidity and mortality [42–45]. The high percentage of patients with NCDs in this study reflect the risk factors of a growing middle class in Nairobi and may be an indication of trends relevant to SSA [5–7]. It is likely then, that in the setting of a low HIV prevalence, NCDs will become the major risk factors for CAP. It is therefore useful to compare results from both high HIV prevalence settings with those from the current study from a private hospital that has a lower percentage of HIV patients but a higher percentage of patients with diabetes and other chronic noncommunicable diseases.

Rhinovirus was found as frequently as influenza, though primarily as a co-pathogen. However, there were four cases in which it was the only agent identified. We therefore included it in our analysis even as we recognized the controversy around its role as a lower respiratory pathogen [46–48]. Numerous other respiratory viruses were also found, either individually or in combination with other viruses or bacteria. It is notable that there were no cases of Mycoplasma identified, indicating that it is not a common cause of hospitalization from CAP.

A meta-analysis by of 31 studies from throughout the world among adults with CAP reported that viral respiratory pathogens were identified in a pooled proportion of 24.5% (range 8–56%) [49]. Among the viruses, influenza was the most frequently reported cause of viral pneumonia folowed by rhinovirus, RSV and human coronaviruses. Similar findings were reported in a systematic review of 27 studies that used PCR for diagnosis of CAP among adults in Europe where they found an average rate of 22% with a range of 6–45% [50]. Again, influenza was the most commonly identified virus, followed by rhinovirus and human coronaviruses. Both reviews reported a high proportion of viral pneumonia with a dual bacterial-viral infection rate of 10% and higher mortality rate among patients with dual infection. The finding of viral isolates in 48% of patients in the current study was toward the upper range of the reported studies.

The low CURB65 and PSI scores in the current study contrast with the high prevalence of complications on admission including AKI, respiratory failure or sepsis (total of 43.6% with at least one of these complications) and requirement for critical care in 31% of patients. Notably, the two mortalities had low CURB65 and PSI scores (PSI of 2 and 3 with CURB65 of 0 and 1). These observations are consistent with other studies that have shown poor ability of these scores to predict prognosis in viral pneumonia patients [51].

This study has a few limitations. First, we recognize that bacterial pathogens in this study were under-recognized, since bacteriologic workup was not part of the study protocol and many patients either had no sputum submitted or had received antibiotics before the sputum was collected. It is also possible that viral etiologies are over-stated, since viral shedding may occur in the absence of symptoms with respiratory viruses [47, 52–54]. However, it is noted that patients with viruses actually had more severe disease as indicated by the need for critical care treatment and the fact that both mortalities were in patients with viruses. Finally, as a single center study with a 10 month duration, we were unable to address seasonality or the presence of rare pathogens.

## Conclusions

In conclusion, this study demonstrated that viral respiratory pathogens play a major role in the etiology and severity of community acquired pneumonia. It is possible that the growth in NCDs such as diabetes and cardiovascular disease in SSA will be accompanied by an increase in community acquired pneumonias with viral pneumonias constituting an important component, especially in non-HIV-infected patients.

## Supplementary Information

**Additional file 1.**

## Data Availability

All data generated or analyzed during this study are included in this published article [and its supplementary information files].
